# Medicine

**Published:** 1915-06

**Authors:** 


### Progress of the Medical Sciences.

### MEDICINE.

**Further notes on the tuberculosis campaign.**[1]—The effects of the war are greatly retarding the work of the tuberculosis campaign: all literary work is carried on at great disadvantage. Many journals have ceased to appear, others are issued at irregular periods, and even the *Tuberculosis Year Book and Sanatoria Annual* (vol. ii.) is of necessity postponed, as it is impossible to get the necessary particulars regarding various schemes, many of the health and tuberculosis officers being away on active service. *The British Journal of Tuberculosis* (October, 1914, vol. viii. No. 4, p. 237) emphasises this fact as follows:—

"Whilst no sympathy and no support will be denied to those who wage war for the protection of the Empire and the maintenance of world-wide liberty, it is essential that the medico-sociological needs of the nation should not be neglected. . . . Judging from experience in other times of national distress, it is safe to prophesy that there will be a considerable increase in the number of necessitous tuberculous cases at all ages. Among our soldiers and sailors it is probable that the heavy strain, long exposure, and physical ills to which they must be subjected will kindle slumbering foci of tuberculosis. . . ."

For the sake of the civilian population it is essential that the tuberculosis campaign should be maintained in full fighting force. No plans and schemes should be suspended, and the Local Government Board should be encouraged in every way to carry on the work they have initiated, and to which they are pledged.

During recent years there has been a conspicuous fall in the mortality from tuberculosis, and it will be of importance to prevent, in so far as is possible, any rise during this time of trial. Moreover, it is pointed out by Dr. Kelynack (C.O.S. Report, 1915) that we must not overlook the influence of the presence of some 250,000 Belgian refugees amongst us, many of whom are known to be tuberculous.

Dr. Kelynack has been very energetic in doing his level best to keep the flag flying, and in spite of many obstacles has been able to prepare the first volume of the *Year Book of Open-Air Schools and Children's Sanatoria*, a companion volume to the *Tuberculosis Year Book and Sanatoria Annual.*[2] This volume

[1] *Vide* vol. xxxii. p. 63, also p. 179.

[2] John Bale, Sons & Danielsson, London. 1915.

should be of much assistance to officers of health, and to many public authorities, and to all who are engaged in anti-tuberculosis efforts. The open-air sanatorium and school stand as embodiments of new principles in preventive medicine, and have already abundantly justified their existence. For delicate children the open-air education and treatment are accomplishing splendid service, and the various reports have shown how much more common is tuberculosis in infancy and childhood than was formerly supposed, so that questions relating to tuberculosis in early life must not be allowed to slide. The chief medical officer of the Board of Education in his reports for 1911 and 1912 gives information on the question of grants in aid. It has been arranged that a sum of £100,000 shall be ear-marked for grants towards the cost of providing sanatorium schools in England for children suffering from pulmonary or surgical tuberculosis, and further maintenance grants may be paid in respect of children attending sanatorium schools. At present the number of effectively equipped sanatoria for children is woefully inadequate. Various schemes are discussed, and it is hoped (to quote Dr. Walford, of Cardiff) "that those bodies who are directly responsible for the provision of institutional treatment will fully realise the importance of securing an efficient administrative co-ordination between the school clinic, dispensary, laboratory, open-air school, sanatorium and hospital.

The nature and aims of the open-air schools are fully described in the *Year Book* (p. 6), and much information is given on the cases suitable for open-air education.

In this neighbourhood an open-air school, similar to those already existing in other towns, and carried out on lines laid down by the Board of Education, has been established, and we have before us the first Annual Report, 1913–14. The school is situated on the high ground at Knowle, on the south-east slope of a hill looking towards the Mendip Hills.

The report gives an account showing that the school has amply justified its existence. It has already done much towards restoring to health these ailing children who were first admitted, many have recovered sufficiently to return to ordinary schools, others are gaining strength daily, and during the first three months the average gain in weight at the fortnightly weighings was shown to be $1\frac{1}{4}$ lb. per child. The daily routine of the school is described in this report, 75 names are on the books, and 108 have passed through during the year. The average stay has been six and a half months.

The **duties of the tuberculosis officer,** as laid down by the Local Government Board, were indicated in our vol. xxxii, p. 64. Since that time Dr. Leonard Crossley[1] has indicated

[1] *Brit. J. of Tub.*, April, 1914, viii. 105.

how these dispensaries are working in the county of Wiltshire. He writes: "The duties centre round the dispensaries, the routine of which, as devised by Sir Robert W. Philip, is now well known. The aim of all this care is that no case of tuberculosis shall remain undetected and uncared for in the community, and that the right sort of treatment shall be provided for each case. . . ." "Being appointed by the County Council, and being on the staff of the County Medical Officer of Health, the County Tuberculosis Officer is brought into close relation with the general public health work of the county, and this is a great advantage. He has the help and advice of the County Medical Officer of Health, and the assistance of his clerical staff. He is informed of all cases of tuberculosis notified, and is able to report all cases of overcrowding and defective sanitation." The tuberculosis officer for Wiltshire writes that he considers that "the Insurance Act as regards tuberculosis is certainly a success. The class of patient who used to go to Winsley Sanatorium paying fifteen shillings a week has now ceased to exist, as if insured they are sent by the Insurance Committee, and if not insured by the County Councils or Boroughs free of cost. Patients used to have to wait for months for the twelve subscribers' beds; now these are reduced to four, they are often not filled, and there is no waiting list. It is quite the exception for patients in Wiltshire to have to wait for three or four weeks. The dispensaries are in full working order and doing good. All tuberculosis patients are kept under observation continuously. After leaving the sanatorium they attend the dispensaries, their progress is watched, and so relapses are prevented. Then again many cases are being sent by their own doctors for aid in early diagnosis, so that they are discovered earlier than formerly. Patients are visited in their own homes by the tuberculosis officer, the dispensary nurses are available, and so their homes are supervised and improved, whilst other members of the family are examined. Shelters are also provided, and this often adds an extra bedroom to an overcrowded house." Until the hospitals for advanced cases are finished it is hard to avoid sending such cases to the sanatorium; such cases ought really to go to the hospital first, and if improving should then be transferred to the sanatorium. Such evidence as the above, from one of our most experienced sanatorium resident officers, is of much interest.

The Charity Organisation Society has warmly supported from the first the adoption of what is known as the dispensary system. Experience has shown that tuberculosis among working people has little chance of success unless it begins and ends with the home. Much information on this point is given by Dr.

Kelynack in his articles published in the *Annual Charities Register* of recent years.

From early days[1] the Charity Organisation Society has recognised how intimately the tuberculosis problem is associated with many branches of charitable work. Tuberculosis is not only a cause of long suffering and loss to the afflicted individual, but it oftentimes entails widespread poverty, deprivation and distress to dependents and associates. Tuberculosis is the cause of from one-fourth to one-third of all deaths among adults at the home-making and child-rearing period of maturity. In homes and orphanages for necessitous children something like 25 per cent. of the children have lost one or both parents from tuberculosis. It must also be remembered that tuberculosis is the most fruitful cause of such delicacy and crippling as commonly leads to dependency or entails assistance from charitable sources. The Charity Organisation Society has, therefore, been compelled to give special attention to the consideration of medico-sociological and economic aspects of tuberculosis. An account of the evolution of the work of the Charity Organisation Society in regard to the anti-tuberculosis movement is given in Mrs. Bosanquet's recent work.[2]

It is pointed out that the greatest single influence in the spread of the disease, namely, contagion through ignorance of the mode of spreading, having been practically eliminated, the subsequent progress towards the abolition of tuberculosis has become slower. It was in 1882 that Koch[3] discovered the bacillus of tubercle and definitely established the contagiousness of the disease.[4]

Dr. Thomas J. Mayo[5] points out that the effect of preventive measures began to be felt in 1899 or 1900. The earliest move of the kind was taken by New York in 1896, Boston in 1900, Philadelphia and Chicago in 1902.

Before 1900 the decline in the death-rate had been irregular, and not very persistently downward. Then it dropped rapidly, and it must be more than coincidence that this rapid drop corresponds so exactly with the period when the contagion was definitely established, and people began to use ordinary care in their daily contact with consumptives. Since 1895 the death-rate has still tended persistently downward, though at a slower pace. The diminution so far noted is chiefly amongst persons in the middle and upper walks of the social strata. It is the general experience that persons of normal intelligence and morals seldom succumb to the ravages of this disease, provided

[1] *Annual Charities Register and Digest.* Charity Organisation Society. 1914.

[2] Bosanquet, *Social Work in London*, 1869 *to* 1912. Murray. 1914.

[3] *Med. Rec.*, lxxxvii. 356. [4] Vide *Bristol M.-Chir. J.*, i. 1883.

[5] *Med. Rec.*, March 20th, 1915.

they are in a financially fit condition to carry out the necessary hygienic regime. The lower or submerged tenth has not been successfully reached. The further reduction of the death-rate is inseparable from the problem of poverty and defective hygiene.

**Poverty and Tuberculosis.**—A booklet published by the New York Association for Improving the Condition of the Poor (105 E. 22J Street, New York City, No. 84) giving the results of two years of the *Home Hospital Experiment* demonstrates three things :—

1. It is possible to treat families in which one or more members are afflicted with tuberculosis by keeping the family together in their own individual home without danger to other members of the family.

2. The results of training patients in their own homes under satisfactory conditions of living with adequate medical supervision compare favourably with results secured by removing the patient from his home and treating him in a sanatorium or other special institution for tuberculosis.

3. In the case of a family in which there are combined tuberculosis and poverty, care of the family as a unit by the Home Hospital method costs less than to break up the family, as is done under other methods of treating families with tuberculosis.

This record of the first experiment along such lines is of much interest and valuable for further efforts of the kind.

The great divergence of opinion as regards tuberculin treatment is still much in evidence. Majors Cochrane and Sprawson,[2] of the Indian Medical Service, have given a good account of their experience. They believe that "the ultimate aim of tuberculin treatment is to produce immunity to the infection by pure tuberculin, by the slow and steady advance of dosage, spread over a very long period. That this result may be achieved in the long run has been fairly well proved, and although by far the larger proportion of cases can only be said to be benefited, still there is reason to look forward to improvements, both in the theory and practice of inoculation, which may lead to a much greater percentage of success in time to come."[3]

Many forms of arthritis, including tuberculous affections, are benefited materially by heliotherapeutics,[4] and the method

[1] *Johns Hopkins Hosp. Bull.*, vol. xxvi., No. 290, p. 123.

[2] Cochrane and Sprawson. *A Guide to the use of Tuberculin*, 1915. London : John Bale, Sons & Danielsson. 5s. net.

[3] Reviewed in *B. M. J.*, 1915, vol. i. 1007.

[4] Jaubert, *La Pratique Héliothérapeutique.* Paris : Baillière et Fils, 1915.

seems to be particularly valuable for children and young subjects generally.

Few of those who advocate heliotherapy have been so enthusiastic and convinced as Rollier, of Leysin (vide *Year Book*, p. 114). He has long urged the value of sunlight in surgical tuberculosis, especially in selected altitudes, the influence being chiefly due to the ultra-violet rays which are the cause of skin pigmentation. The open-air school classes at Leysin under heliotherapeutic influences and continuous exposure to sun and air have a markedly beneficial effect on tuberculous abscesses and sinuses. Great importance is attached to the bronzing of the skin as an index of improvement, and it is said that no pigmented skin becomes the seat of acne in tuberculosis. It is obvious that good results cannot be expected from this method in this country and in winter.

**Asphyxiating Gases.**[1]—The composition of the asphyxiants used by the Germans in forcing back the French and English lines has led to much conjecture. It has been surmised that the gas used was carbon monoxide, but as this gas is slightly lighter than air, it would diffuse too rapidly to accomplish the object of the enemy. Later reports show that the gas is a true asphyxiant, such as sulphur dioxide, chlorine or a mixture of the two; these satisfy the requirements of being more than double the weight of air, and so would remain near the ground and only diffuse slowly. It has been said that bromine gas and formaline have also been used largely, but we must await the report of Dr. J. S. Haldane before we get any authoritative information on the subject. The efficacy and value of inhaled vapours are a much debated question when used for therapeutic purposes, but of the efficacy of the German gas to asphyxiate their enemy there has been more than ample proof. From further evidence we learn that there can be little doubt that chlorine or bromine was the chief agent employed, whilst shells containing other irritant poisons were also used.

"Both chlorine gas and bromine vapour, when present to the extent of 5 per cent. in air, rapidly cause death by suffocation by acting on the mucous linings of the nose, throat and lungs, so causing acute inflammation; but bromine poisoning is generally distinguishable by the skin of the victim being turned yellow, and the intense action on the eyes, which is much greater than with chlorine. The Germans have an unfailing source of bromine in the crude carnallite worked at Strassfurt for the production of potassium chloride, but it will probably be found that besides such obvious asphyxiants as chlorine, bromine and sulphur dioxide, they have also employed compounds of a more complex character."[2]

[1] *Nature*, April 29th, p. 234. [2] *Nature*, May 6th, 1915, xcv. 267.

The Germans have apparently contrived to dispatch the gas by means of shells, and one officer's experience was that in the bursting of the shells a green gas was developed, and before he and his comrades had a chance of moving they were inhaling the poisonous gas, and a moment later were struggling terribly for breath. . . . He had no doubt that the gas in the shells and the curtain of "creeping gas" were of the same nature. [1]

### Reviews of Books.

[1] Vide *Lancet*, May 15th, 1915, i. 1036.

